# A comprehensive analysis of clinical trials in pancreatic cancer: what is coming down the pike?

**DOI:** 10.18632/oncotarget.27727

**Published:** 2020-09-22

**Authors:** Erryk S. Katayama, Jonathan J. Hue, David L. Bajor, Lee M. Ocuin, John B. Ammori, Jeffrey M. Hardacre, Jordan M. Winter

**Affiliations:** ^1^Department of Surgery, Case Western Reserve University School of Medicine, Cleveland, Ohio, USA; ^2^Department of Surgery, University Hospitals Seidman Cancer Center and Case Comprehensive Cancer Center, Cleveland, Ohio, USA; ^3^Department of Medicine, Case Western Reserve University School of Medicine, Cleveland, Ohio, USA; ^4^Division of Hematology and Oncology, University Hospitals Seidman Cancer Center, Cleveland, Ohio, USA; ^5^Division of Hepatobiliary and Pancreatic Surgery, Atrium Health, Charlotte, North Carolina, USA

**Keywords:** pancreatic cancer, pancreatic ductal adenocarcinoma, clinical trial, novel treatment, drug development

## Abstract

Objective: Pancreatic cancer is the most aggressive common cancer and is desperately in need of novel therapies. Unlike many other common cancers, there have been no new paradigm-changing therapies in the past 40 years beyond multi-agent chemotherapy. In this study, we perform the first comprehensive analysis of the current clinical trial landscape in pancreatic cancer to better understand the pipeline of new therapies.

Materials and Methods: We queried https://clinicaltrials.gov/ for registered pancreatic cancer clinical trials. Studies were curated and categorized according to phase of study, clinical stage of the study population, type of the intervention under investigation, and biologic mechanism targeted by the therapy under study.

Results: As of May 18, 2019, there were 430 total active therapeutic interventional trials testing 590 interventions. The vast minority of trials (*n* = 37, 8.6%) are in phase III testing. 189 (31%) interventions are immunotherapies, 69 (11%) target cell signaling pathways, 154 (26%) target cell cycle or DNA biology, and 35 (6%) target metabolic pathways. Of the late phase trials, only 14 are currently testing novel interventions. Rather, 23 phase III trials examine new ways to deliver existing FDA-approved drugs, procedures, or pain management.

Conclusions: A large number of novel therapeutic strategies are currently under investigation. They include a broad range of therapies targeting diverse biologic processes. However, only a small number of novel therapies are in late-stage testing, suggesting that future progress is likely several years away, and dependent on the success of early-stage trials.

## INTRODUCTION

Pancreatic ductal adenocarcinoma (PDAC) is the most aggressive of the common cancers. Five-year survival has only marginally improved over the past 45 years (1975 to 2020, 3% to 9%) [1, 2]. To date, there are few drugs which have been approved by the United States Food and Drug Administration (FDA) for treatment of PDAC. With the exception of two targeted agents, which exhibited minimal or no overall survival benefit (erlotinib and olaparib) [3, 4], standard treatments are limited to conventional chemotherapies. Approved multi-agent chemotherapy combinations include a) gemcitabine plus nab-paclitaxel, and b) folinic acid, fluorouracil, irinotecan, and oxaliplatin (FOLFIRINOX) [5, 6]. These cocktails have become the standard first-line choices, and confer a median overall survival benefit of around 2–4 months over gemcitabine monotherapy in patients with advanced disease. Liposomal irinotecan rounds out the standard options. The nanoparticle-encapsulated chemotherapeutic is approved in combination with 5-fluorouracil (5-FU) as a second line treatment, and offers a survival benefit in this combination of just two months over 5-FU alone [7].

Thus, there have been no paradigm-shifting advances beyond combination chemotherapy in the pancreatic cancer field over the past two decades. This contrasts with many other common cancers, which have benefited from impactful targeted therapies (e.g., trastuzumab for breast cancer, imatinib for chronic myelogenous leukemia) or immunotherapy (e.g., checkpoint inhibitors for melanoma and lung cancer) [8–10]. In light of the fact that PDAC is especially aggressive, there is an urgent need to develop novel and effective treatments.

The National Cancer Institute (NCI) is the principal funding arm of the National Institutes of Health (NIH) dedicated to funding cancer research. The institute allocates roughly $6 billion annually to cancer research, and just over $100 million is dedicated to study pancreatic cancer [11]. This amounts to a running total for pancreatic cancer research nearing $2 billion appropriated over the past two decades to improve PDAC survival. Fortunately, spending is on the rise. NIH-directed distributions reached $153 million and $178 million in 2016 and 2017 respectively [11]. Other agencies and organizations like the Pancreatic Cancer Action Network (PanCAN), American Cancer Society, and the Department of Defense contribute significantly to this mission, likely adding more than $20 million per year in totality [12–14]. Beyond this, industry-sponsored research has contributed tens of millions over and above these amounts.

In 2011, PanCAN declared a “vision of progress” with the goal of doubling survival by 2020 [12]. Data are certainly mixed on whether the vision was realized. On the positive side, survival achieved in the adjuvant PRODIGE trial is more than twice that observed historically for resected PDAC [15]. However, one cannot attribute substantial progress in survival to any investments into more innovative scientific discoveries. Rather, progress was the result of a combination of old chemotherapies. Along these lines, we have likely neared a survival ceiling for our patients in the absence of new discoveries that target other aspects of cancer biology, due to the additive toxicities of chemotherapeutic combinations.

Patients, family members, primary care providers, and oncologists battling together against pancreatic cancer often consider the same important questions: what new treatments are coming down the pike, and how soon will they arrive? These questions are almost impossible for any one expert to answer reliably; the field is too vast and the number of studies is too great. We provide an attempt at a comprehensive answer in this study.

Clinical trials represent the furthest steps in the development of new therapies, and therefore represent the best source to examine the question of promising therapies. Herein, we present the complete compendium of ongoing clinical trials in pancreatic cancer to profile the landscape of experimental therapies furthest along the development pipeline. This analysis seeks to organize, analyze, and summarize interventions under current investigation. In doing so, we determine the number of ongoing trials and characterize the full breadth of therapies being studied. This work is necessary to better anticipate the timeframe for novel therapies against pancreatic cancer to reach market. More importantly, this 20,000-foot view provides a foundation to discuss optimal resource allocation, with a principal goal to accelerate the pace of innovation, with an eye towards improving patient outcomes.

## RESULTS

### Overview of all trials

**Figure 1:**
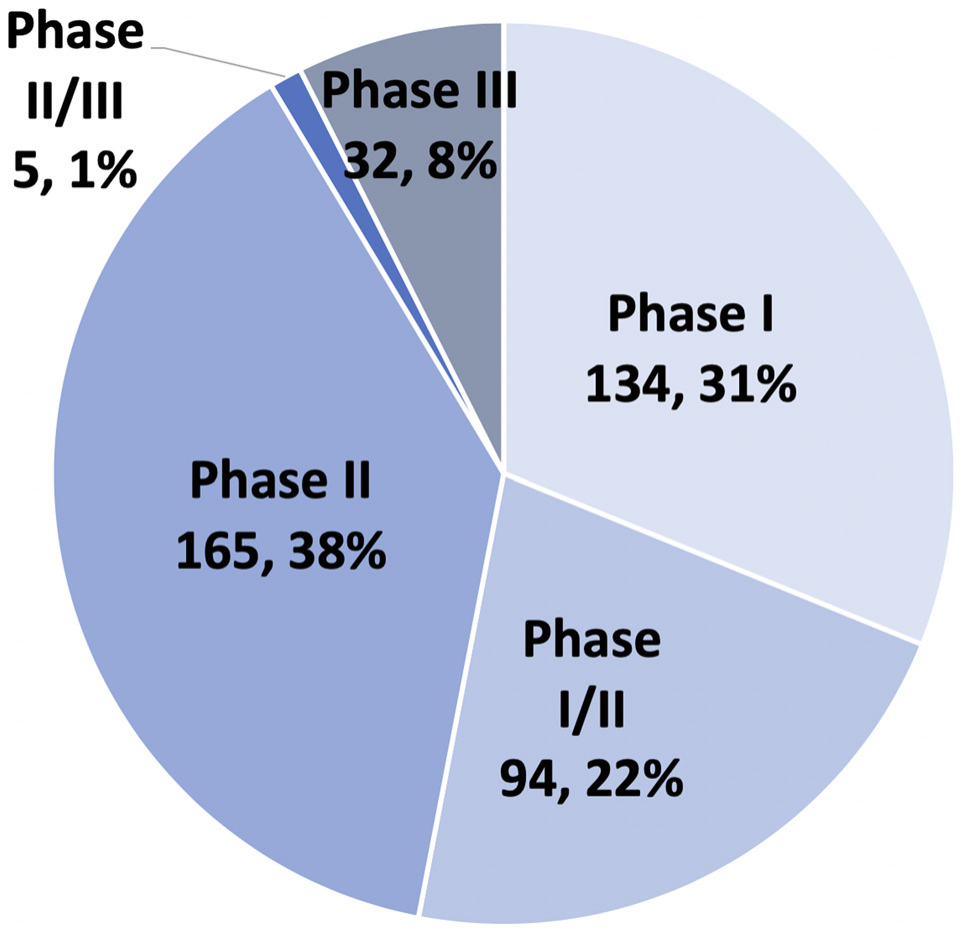
Clinical phase of 430 PDAC trials.

As of May 18, 2019 (the locked-in date), there were 430 registered interventional trials focused on pancreatic cancer. The distribution of these trials are as follows: 134 (31%) in phase I testing, 94 (22%) in phase I/II testing, 165 (38%) in phase II testing, 5 (1%) in phase II/III testing, and 32 (8%) in phase III testing (Figure 1). The majority of the 430 distinct trials enroll patients with metastatic pancreatic cancer (*n* = 265, 62%), and 135 focus on patients with localized PDAC (31%). The latter subgroup is closely split between resectable and unresectable PDAC (Table 1). 163 out of 430 (38% of the trials) are first line studies and 168 (39%) investigate second or later lines of therapy. The remaining studies (*n* = 99, 23%) do not specify.

**Table 1:** Patient populations by clinical stage of disease and line of treatment, for 430 trials

Stage of Disease		
**Advanced/Metastatic**	**265**	**62%**
**Localized**	**135**	**31%**
Resectable	60	14%
Borderline	19	4%
Unresectable	46	11%
Any	10	2%
**Unspecified**	**30**	**7%**
**Line of Therapy**		
**First**	**163**	**38%**
**Second**	**168**	**39%**
**Unspecified**	**99**	**23%**

### Therapeutic categories

**Figure 2:**
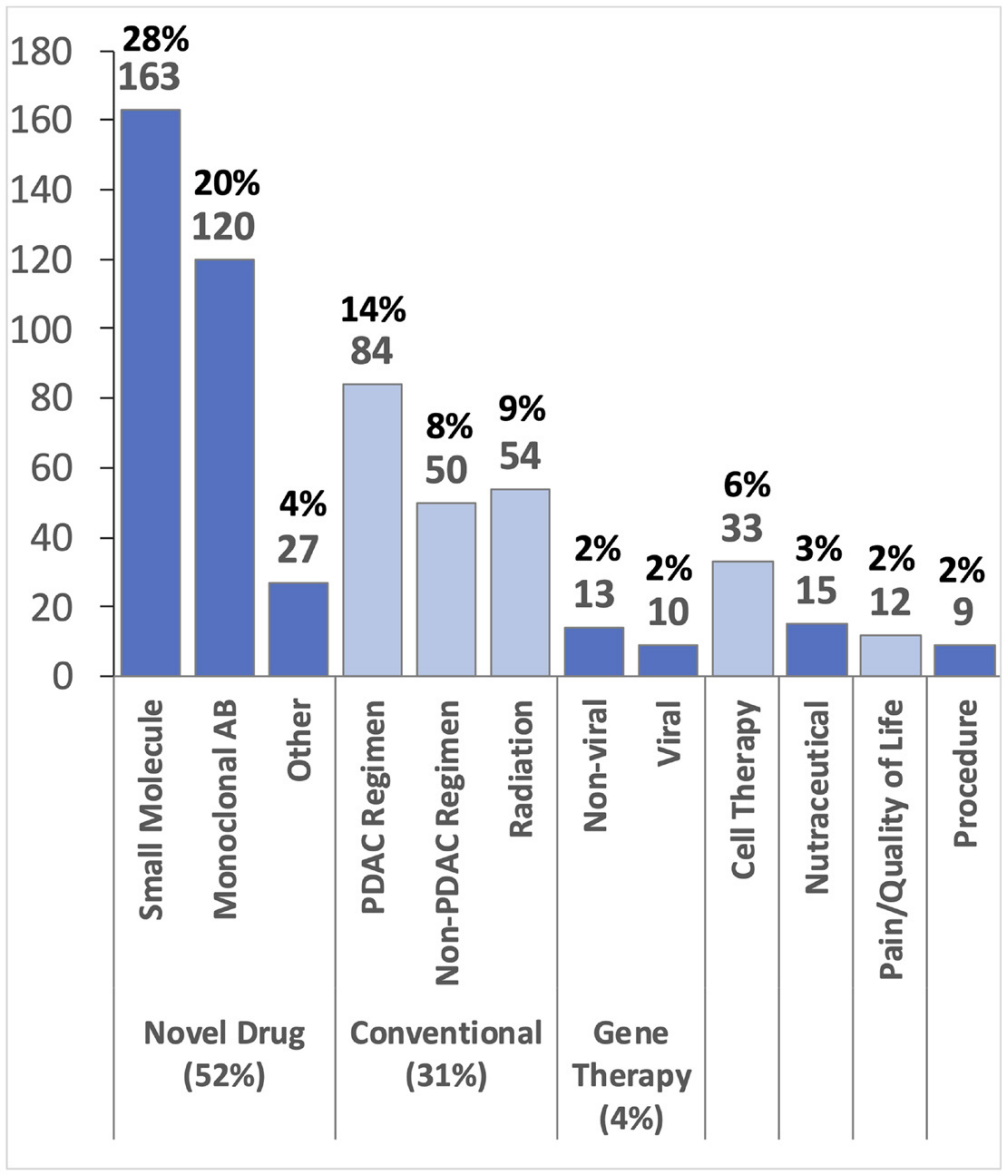
Classification of 590 different therapeutic interventions. Many of the 430 trials investigate multiple interventions. Abbreviations: Antibody (AB), Pancreatic Cancer (PDAC).

A total of 590 therapeutic interventions are investigated across these 430 trials, and span a wide range of therapeutic categories (Figure 2). 310 of the 590 (52%) test novel drugs or pharmacologic agents, including 163 (28% overall and 53% of drug studies) small molecule drugs and 120 (20% overall and 39% of drug studies) monoclonal antibodies. Remaining studies include miscellaneous pharmacologic agents, such as recombinant proteins or other biologics. 188 (31%) therapeutic agents under investigation are considered conventional or existing drug therapies. Trials integrating conventional therapies include 84 (14%) that test novel combinations or delivery methods of established pancreatic cancer drugs, 50 (8%) trials testing FDA-approved therapies already in use for other cancer types (i.e., non-pancreatic cancer), and 54 (9%) radiation-based interventions. 23 (4%) gene therapy trials are enrolling, and the majority use non-viral vectors. Remaining intervention strategies include 33 (6%) cellular therapy trials, 15 (3%) nutraceutical trials, 9 (2%) trials assessing technique or procedure-focused interventions, and 12 (2%) trials focus on pain management or quality of life.

### Biologic targets

In total, there are 515 therapies targeting some aspect of PDAC biology through pharmacologic, cell-based, viral-based, or other systemic delivery systems. We classified these therapies according to their biologic targets to map this mechanistic landscape (Table 2). In total, 189 (37% of pharmacologic interventions) immunotherapies dominate the clinical trial landscape. These studies include 73 checkpoint inhibitor trials (14% of total pharmacologic interventions), 33 adoptive cell transfer trials (6.5%), 10 oncolytic virus studies (2%), 28 cancer vaccine trials (5.5%), and 45 other miscellaneous immune or cytokine intervention trials (9%).

**Table 2:** 515 pharmacologic interventions classified by mechanism of action

Interventions by Mechanism		% (*n* = 515)
**Immune system**	**189**	**37%**
Checkpoint Inhibitor	73	14%
Adoptive Cell	33	6.5%
* T-Cell*	*19*	*3.5%*
* Other*	*14*	*3%*
Oncolytic Virus	10	2%
Cancer Vaccine	28	5.5%
Other Immune	45	9%
**DNA and Cell Cycle**	**154**	**30%**
**Cell Signaling**	**69**	**13%**
RTKs	20	4%
KRAS/RAF/ERK/MEK	15	3%
PI3K/AKT/mTOR	14	3%
JAK-STAT	8	1%
FAK/SRC	6	1%
Misc. (HH, WNT, Hsp90, GSK-3, TGFβ)	6	1%
**Metabolism**	**35**	**7%**
**Angiogenesis**	**17**	**3%**
**Hormone Receptors**	**15**	**3%**
**Apoptosis Specific**	**15**	**3%**
**Target Symptoms**	**11**	**2%**
**Metastasis/Invasion**	**7**	**1%**
**Unknown Mechanism**	**3**	**< 1%**
**Total**	**515**	**100%**

Abbreviations: receptor tyrosine kinase (*RTK*), miscellaneous (misc.), hedgehog (*HH*), heat shock protein 90 (*Hsp90*), glycogen synthase kinase 3 (*GSK-3*), transforming growth factor beta (*TGFβ*).

DNA and cell cycle targeting drugs comprise the next largest mechanistic class. These trials include the classic chemotherapeutic agents (*n* = 154, or 30%). 69 (13%) therapies under investigation target cell signaling. The most common pathways targeted in this group include receptor tyrosine kinases such as epidermal growth factor receptor (EGFR), platelet-derived growth factor receptor (PDGFR), vascular endothelial growth factor (VEGF), AXL, and KIT (*n* = 20, 4% overall). Additional target pathways include KRAS/ERK/MEK (*n* = 15, 3%), PI3K/AKT/mTOR (*n* = 14, 3%), JAK-STAT (*n* = 8, 1%), and FAK/SRC (*n* = 6, 1%). Less common cell signaling targets include hedgehog (HH), WNT signaling, heat shock protein 90 (Hsp90), glycogen synthase kinase 3 (GSK-3), and transforming growth factor beta (TGFβ). Other biologic processes under therapeutic study are cellular metabolism (*n* = 35, 7%), angiogenesis (*n* = 17, 3%), hormone receptors (*n* = 15, 3%), apoptosis (*n* = 15, 3%), and metastasis and invasion (*n* = 7, 1%). Additionally, therapeutic trials are underway to treat biologic sequelae of PDAC, as opposed to the cancer itself, including thrombosis (*n* = 5, 1%), infections (*n* = 3, < 1%), and digestive enzyme supplementation (*n* = 3, < 1%).

### Phase III trials

We further explored phase III trials, since these have the potential to change the treatment paradigm in the near-term if successful (Table 3). Out of 37 phase II/III and III trials testing treatments against PDAC, only 14 are testing novel interventions in the PDAC space. Some of the 14 novel therapeutic trials are studying interventions that have already shown efficacy in other cancers (*n* = 3) while two trials are repurposing existing FDA-approved non-cancer drugs (METRICS and clopidogrel). The remaining trials (*n* = 9) examined interventions that are novel to both PDAC and other cancers. The remaining phase III trials are either manipulating chemotherapeutics already used to treat PDAC or other cancer types (*n* = 13), studying an intervention related to technical treatment strategies (*n* = 7), or focused on pain management (*n* = 3).

**Table 3:** A compendium of 37 phase III trials by type, mechanism, and impact score

Clinical Trials Identifier	Intervention	Type	Mechanism	Endpoint	Impact Score
NCT03377491	**Tumor Treating Fields** + Gemcitabine + Nab-Paclitaxel (PANOVA-3)	Device (Electric Currents)	Cell Cycle (Cytoskeleton)	Survival	15
NCT03504423	**Devimistat (CPI-613)** + mFFX (AVENGER 500)	Drug - Small Molecule	Mitochondrial Metabolism	Survival	13
NCT03126435	**EndoTAG-1** + Gemcitabine	Drug - Small Molecule	Cationic Liposomal Paclitaxel	Survival	11
NCT03766295	**Mastinib** + Gemcitabine	Drug - Small Molecule	Cell Signaling (RTKs c-Kit, PDGFR, FGFR3)	Survival	10
NCT02184195	**Olaparib** (POLO)	Drug - Small Molecule	DNA and Cell Cycle (BRCA)	Survival	9
NCT02948309	**Mistletoe Extract**	Drug - Other	Immune System	Survival	9
NCT01926197	mFOLFIRINOX +/− SBRT in Locally Advanced PDAC	Conventional - FDA Chemoradiation	—	Survival	9
NCT01077427	**Hyperthermic** Gemcitabine and Cisplatin (HEAT)	Conventional – FDA Approved Chemo	—	Survival	8
NCT03251365	**Hyperthermic Intra-abdominal Chemotherapy (HIPEC)**	Conventional - FDA Approved Chemo	—	Survival	8
NCT03649035	**HybridTherm Probe (HTP)**	Ablation Device (Cryothermal)	—	Survival	8
NCT03398291	**Simultaneous Resection** of Pancreatic Cancer and Liver Oligometastasis	Procedure	—	Survival	8
NCT03721744	**2nd Line Napabucasin** + Gemcitabine + Nab-Paclitaxel	Drug - Small Molecule	Stemness (STAT3)	Survival	7
NCT02923921	**Pegilodecakin (AM0010)** + FOLFOX	Drug - Other	Immune System	Survival	7
NCT03468335	**2nd line Irinotecan Liposomal Injection (Onivyde)**	Conventional - FDA Approved Chemo	—	Survival	7
NCT01964430	**Adjuvant Gemcitabine + Paclitaxel** vs Gemcitabine (APACT)	Conventional - FDA Approved Chemo	—	Survival	7
NCT02993731	**1st Line Napabucasin** + Gemcitabine + Nab-Paclitaxel	Drug - Small Molecule	Stemness (STAT3)	Survival	6
NCT03610100	**Acelarin**	Delivery - PC Chemo	Prodrug (Gemcitabine)	Survival	6
NCT03257033	**Intra-arterial Gemcitabine (RenovoCath)** vs Intra-venous Gemcitabine	Conventional - FDA Approved Chemo	—	Survival	6
NCT02201381	**Metformin + Atorvastatin + Doxycycline + Mebendazole** (METRICS)	Drug (s) - Small Molecule	Metabolism	Survival	6
NCT01954992	**Glufosfamide** vs 5-FU	Delivery – non-PC Chemo	Ifosfamide + Glucose (delivery)	Survival	6
NCT01013649	Adjuvant Gemcitabine +/− **Erlotinib +/**− **Radiation + Capecitabine/Fluorouracil**	Conventional - FDA Approved Chemo	—	Survival	6
NCT02539537	**Neoadjuvant FOLFIRINOX** vs Gemcitabine (NEOPAN)	Conventional - FDA Approved Chemo	—	Survival	5
NCT02853474	**Early Palliative Care** (metastatic)	Quality of Life	—	Quality of Life	5
NCT03472833	**High-dose Vitamin D**	Nutraceutical	Unknown	Quality of Life	4
NCT01827553	**Chemoradiation** vs. Chemotherapy alone in Locally Advanced PDAC (CONKO-7)	Conventional - Radiation	—	Survival	4
NCT02195232	**Isoquercetin**	Drug - Small Molecule (Anti-thrombosis)	Anti-thrombus (PDI)	Sequelae	4
NCT02404363	**Clopidogrel**	Drug - Small Molecule	Growth and Metastasis (Platelets)	Survival	3
NCT02919787	**Neoadjuvant** Chemotherapy	Conventional - FDA Approved Chemo	—	Survival	3
NCT02172976	**Neoadjuvant + Adjuvant FOLFIRINOX** vs Adjuvant Gemcitabine vs (NEPAFOX)	Conventional - FDA Approved Chemo	—	Survival	3
NCT02506842	**2nd line Gemcitabine + Nab-paclitaxel vs FOLFOX**	Conventional - FDA Approved Chemo	—	Survival	3
NCT02457156	**Blumgart Anastomosis vs Cattell-Warren Anastomosis**	Procedure	—	Complication	3
NCT02514928	**Resection of Nerve Plexus on Right Half of Celiac and SMA** Associated With Extended Pancreatoduodenectomy	Procedure	—	Complication	3
NCT02871804	**Combined vs Separated Resection of Splenic Vein**	Procedure	—	Survival	3
NCT03269994	**Piperacillin-tazobactam or Cefoxitin** post-surgery	Drug - Small Molecule (Post-procedure)	Antibiotic	Infection	2
NCT03434678	**Epidural**	Pain Management	—	Pain	2
NCT02340728	**Radiofrequency Ablation** + Self Expandable Metal Stents	Ablation + Device	—	Sequelae	0
NCT02349412	**Early Palliative Care**	Quality of Life	—	Quality of Life	0

The novel intervention being tested is bolded.

The 37 trials are detailed in Table 3, and ranked according to a novel calculated impact score (IS, see methods). This sorting estimates the most promising therapies that are far down the drug development pike, using criteria that attempt to minimize subjectivity and prioritize the potential survival benefit. The top three are profiled in greater detail below, and are considered the most promising (IS ≥ 11) using these objective criteria. The ranking may not follow an order that reflects conventional thinking; indeed, we intended to deprioritize and ignore intuition or subjectivity. The impact score point breakdowns are detailed in Supplementary Table 1. For validation, we retrospectively scored two different landmark trials using our scoring system. The PRODIGE trial which compared the administration of FOLFIRINOX to gemcitabine monotherapy in patients with metastatic pancreatic cancer [5] received a score of 13/18. In patients with metastatic melanoma, the administration of gp100 with or without ipilimumab [16] received a score of 14/18. We assigned these scores as if they were evaluated at the time of patient accrual (Supplementary Table 2).

The highest scoring trial (15/18 points) in the list of PDAC phase III trials is the PANOVA-3 (NCT03377491) trial. This trial investigates the use of Tumor Treating Fields (TTF) in locally advanced PDAC patients. TTF are electrical currents that prevent mitosis through disruption of the spindle apparatus and cytokinesis. In pre-clinical studies, these fields cause death of rapidly dividing cancer cells, including glioblastoma (for which it is FDA approved) and PDAC [17, 18]. The preceding PANOVA-2 trial demonstrated that TTF combined with gemcitabine significantly improved median progression free survival (PFS) (8.3 months vs. historical control of 3.7 months) and median overall survival (OS) (14.9 months, as compared to 6.7 months in the historical control) [17].

The next highest scoring trial (13/18 points) is AVENGER 500 (NCT03504423), which is investigating the use of CPI-613, a first-in-class mitochondrial inhibitor targeting pyruvate dehydrogenase and ketoglutarate dehydrogenase. In this phase III trial, the drug is added to a modified FOLFIRINOX backbone. The addition of CPI-613 in the preceding phase I trial effectively doubled the historical objective response rate from 31% to 61% with an associated PFS of 9 months and a median OS of 19 months (compared to 11.1 months historically for FOLFIRINOX). Minimal added toxicity was observed [19, 20].

NCT03126435 (11/18 points) examines the use of EndoTAG-1 in combination with gemcitabine, as a second-line treatment for advanced PDAC. EndoTAG-1 employs a cationic liposome membrane to selectively deliver paclitaxel to the negatively charged tumor endothelium, while hopefully minimizing systemic toxicities. A previous phase II trial demonstrated that multiple doses of EndoTAG-1 (11 mg/m^2^, 22 mg/m^2^, and 44 mg/m^2^) combined with gemcitabine had a survival benefit compared to gemcitabine historical control. Specifically, the trial determined that 22 mg/m^2^ provided the best risk-benefit ratio while increasing PFS from 2.7 to 4.6 months and median OS from 6.8 to 8.7 months in advanced stage PDAC patients [21].

Since the locked-in date of the study analysis, 7 of the 37 phase III trials have been completed or terminated early, which has thinned the prospective pool of promising phase III trials down 30. None of these matured trials have drastically altered the treatment paradigm. The POLO trial is the fifth highest rank trial by impact score and serves as a solid reference point. This was indeed a positive trial, and the phase III study led to the FDA approval of olaparib in 2019, making it the first pancreatic cancer drug to pass this regulatory threshold in more than four years. Olaparib is a targeted inhibitor of poly-ADP ribose polymerase (PARP). In patients harboring PDAC with *BRCA* mutations, the drug significantly increased PFS from 3.8 months to 7.4 months as maintenance therapy (after platinum-based palliative therapy) when compared to placebo. Unfortunately, there was no difference in overall survival at interim analysis (18.9 months vs 18.1 months) or quality of life [4].

The CanStem111P trial (NCT02993731) tested napabucasin, a cancer stemness inhibitor, in conjunction with gemcitabine and nab-paclitaxel in metastatic PDAC. The combination failed to improve OS and was terminated due to futility at interim analysis [22]. Other mature trials round out the pipeline story with a similar narrative. The SEQUOIA trial (NCT02923921) testing pegilodecakin (pegylated IL-10) plus FOLFOX (folinic acid, fluorouracil, oxaliplatin) as second-line treatment for metastatic PDAC failed to meet OS endpoints [23]. The PANCREADOGREL trial (NCT02404363) testing clopidogrel as an anti-platelet drug in advanced disease sought to slow down tumor progression and metastasis formation. The trial was prematurely terminated due to recruitment issues [24]. The ACELARATE trial (NCT03610100) examined acelarin, a gemcitabine prodrug, as a first line monotherapy for metastatic PDAC. The trial was suspended due to futility and toxicities at the interim analysis [25]. Of the remaining two completed trials, one was a palliative study designed to improve quality of life in patients with incurable disease while the other used radiofrequency ablation and stents to relieve biliary obstruction [26, 27].

## DISCUSSION

Cancer mortality has improved across numerous subtypes over the past three decades, due largely to improved cancer screening and decreased tobacco use [1]. The improvement trend in recent years is also attributable to progress in cancer therapies. However, targeted drugs and immunotherapies have yet to consistently impact patients with PDAC. Instead, progress in PDAC consists of incremental advances with chemotherapy over the past 23 years (Table 4).

**Table 4:** FDA approved PDAC chemotherapies with published survival benefits in palliative and adjuvant settings

Year (Palliative)	Intervention	Palliative Survival (mo.)	Adjuvant Survival Benefit (mo.)
1962	Fluorouracil	4.41	11 vs. 20
1997	Gemcitabine	4.41 vs. 5.65	20.2 vs. 22.8
2005	Erlotinib + Gemcitabine	5.91 vs. 6.24	No benefit
2011	FOLFIRINOX	6.8 vs. 11.1	35.0 vs 54.4
2013	Nab-paclitaxel + Gemcitabine	6.7 vs. 8.5	36.2 vs. 40.5^*^
2015	Liposomal Irinotecan	4.2 vs. 6.1	—
2019	Olaparib	18.1 vs. 18.9^*^	—

Year of publication refers to the palliative chemotherapy trials for advanced PDAC. (^*^) denotes data taken from interim analysis of an ongoing clinical trial.

In 1997, gemcitabine replaced 5-FU as the cornerstone of pancreatic cancer chemotherapy in patients with advanced disease because of a 1.25-month survival benefit [28]. Ten years later, gemcitabine became the standard in the adjuvant setting (the CONKO-001 trial) with a 2.6-month survival advantage over observation [29]. After more than 30 negative trials, several multi-agent chemotherapy regimens finally proved to be superior in the advanced setting. The first was gemcitabine and erlotinib (10-day survival benefit) [3]. Subsequent examples included gemcitabine and nab-paclitaxel (1.8-month survival benefit) [6] and FOLFIRINOX (4.3-month survival benefit) [5]. In the adjuvant setting, the standard-of-care shifted for a brief time to gemcitabine and capecitabine (ESPAC-4, 3-month benefit) [30]. More recently, adjuvant FOLFIRNOX resulted in a 19-month survival advantage over gemcitabine monotherapy [15]. However, the survival advantages achieved with these regimens are likely near their limit. Novel approaches targeting different aspects of tumor biology are necessary for future leaps in survival outcomes. It is unlikely that additional chemotherapeutics can be added due to overlapping toxicities. As stated above, olaparib represents a lone example of an approved targeted or precision medication, but overall survival is not significantly improved in genetically eligible patients [4]. One could offer pembrolizumab for microsatellite instability high (MSI-H) PDAC patients as another example, but the response rate and outcomes are poor even for this exception, and MSI-H is extremely rare for PDAC (< 1%) [31–33].

These realities raise difficult questions. When will the field experience significant advances beyond chemotherapy? How can these advances be accelerated? Why is pancreatic cancer such an outlier with respect to progress? While PDAC may be inherently different from a biological standpoint, practical considerations likely relate to the economics and logistics of cancer research. Importantly, new perspectives and truths begin to emerge from a better understanding of the PDAC clinical trial landscape.

**Figure 3:**
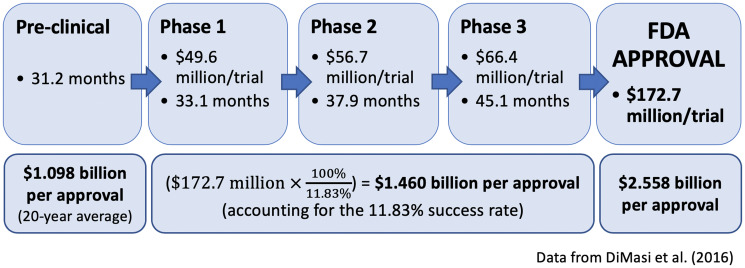
Summary of the time and cost for drug development (modified from DiMasi et al. [2016]). Costs factor in the 11.83% success rate through all three trials.

The drug development process is both expensive and time-consuming. A 2016 study by the Tufts Center for the Study of Drug Development estimated the average time for a novel compound to move from the pre-clinical stage to FDA approval takes approximately eight years [34]. Pre-clinical studies take 31.2 months, but this does not factor target discovery and validation, which often takes a decade or more. Each of the clinical phases of trial testing takes around three to four years. Although DiMasi *et al.* estimates that only about 11% of all drugs that enter clinical testing (excluding pre-clinical testing) actually achieve FDA approval, success in pancreatic cancer is even less likely, with an estimated probability of success near 3% [35, 36]. Each of these phases can cost tens of millions of dollars, and the total cost to develop a drug can exceed $2 billion if you integrate the costs of failed drugs (Figure 3) [34].

Pancreatic cancer funding has increased substantially over time [11, 37]. Twenty years ago, the field was receiving less than $20 million per year for research. The number stabilized at $100 million in the early 2010s. The Recalcitrant Cancer Research Act in 2013 increased that number to almost $200 million annually [37]. Despite this, PDAC is still under-funded. Currently, the NCI allocates $6 billion broadly for cancer research, but only three percent is designated for pancreatic cancer in concept. This reflects cancer incidence rates, but pancreatic cancer is responsible for 7.7% of cancer-related mortalities, and this proportion will likely increase in the coming years [1]. Moreover, many funded studies attributed to pancreatic cancer research in these calculations by NCI funding analytics may not be truly focused on PDAC, as the figures imply. Rather, experts note that pancreatic cancer is only peripherally related in many of these funded studies (e. g., a study on KRAS targeting may be attributed to pancreatic cancer research) [38]. Thus, PDAC may not be receiving as much funding as publicly reported.

The study herein highlights 430 ongoing clinical trials. These data suggest that there is hope in the long-run, but short-run prospects are more challenged. 590 therapies or therapeutic strategies are under investigation, which is a significant number of potential opportunities. Notably, many of the interventions are similar or identical to each other, reflecting potential redundancy in the process. They span numerous therapeutic categories or treatment modalities- including small molecule drugs, antibodies, gene therapies, cell therapies, nutraceuticals, and novel procedural approaches. The therapies target cancer through a broad range of mechanistic strategies, including immunotherapies, cell signaling pathways, metabolic pathways, angiogenesis, hormone receptors, apoptotic signaling, and cell migration. Success in just one of these clinical trials may shift the paradigm within the next five to ten years.

However, just 30 ongoing phase II/III and phase III trials are candidates for FDA approval, if successful. The study presented here highlights that many of these trials look to optimize conventional treatment modalities, such as neoadjuvant trials for localized disease. Many of the trials are looking at symptom endpoints, not cancer-specific survival. In some cases, existing drugs are getting repurposed. Thus, there are just 14 phase III interventional trials testing therapies that have never been tested before in PDAC (not counting the early-staged trials that led to the index phase III trial). Unfortunately, considering historical success rates, the likelihood of drastically improving PDAC survival or changing the treatment paradigm over the next five years is quite low.

Several of the most promising studies are highlighted here. A novel ranking system that factored in compelling early trial data and indicators of novelty suggest that the application electric currents (TTF) to disrupt cancer growth, utilization of a small molecule that targets mitochondria, or usage of nanoparticle-delivered paclitaxel have the most promise among existing phase III trials. Even these, from a biologic standpoint and on the surface, seem unlikely to significantly improve survival. Tumor Treating Fields are a regional therapy, yet PDAC is a disease that is principally systemic in its behavior. The EndoTAG-1 trial seeks to enhance delivery of an old chemotherapeutic. Even these trials seem rather unlikely to substantially improve survival.

There are several notable limitations to our study. Our data only includes trials that are registered on https://clinicaltrials.gov/ under “Pancreatic Cancer”. Trials omitted from our consideration include any unregistered phase I trials, international trials outside of the scope of the United States, and perhaps trials that recruit from a variety of cancers (umbrella or bucket design) that do not specify pancreatic cancer as a target. Another limitation is that the IS system is biased towards survival endpoints. Trials with end-goals other than survival, such as palliative care or quality of life, will score poorly in the IS, despite successfully reaching their end-point. These trials can hold tremendous value in advancing standard of care and their importance should not be down-played. Moreover, while the actual definitions used to score trials are objective, criteria were chosen by the study-authors, which still enables an element of subjectivity to creep into the process.

The majority of PDAC trials are focused immunotherapy (37%), chemotherapy (22%), and radiation (9%). Perhaps diversifying the research landscape and investigating other biologic pathways, such as metabolism or hormone physiology, could increase the chances of success. As Dr. Scott Kern so eloquently put it in an interview on the Pancreatic Cancer Podcast, there is a tendency to follow the herd in research [38, 39]. Following the herd has not yet worked well for PDAC research; immunotherapy and precision therapy have yet to strongly impact this disease. This study should heed some warning that we may be allocating our chips too heavily in these directions. Finally, this compendium focuses on therapeutic studies and not early detection. It is possible that the greatest advance in the future could be the discovery of an effective PDAC biomarker. If we can detect PDAC at the PanIN 3 (carcinoma *in situ*) stage, therapeutic trials of invasive cancer become inconsequential.

## MATERIALS AND METHODS

The registry on https://clinicaltrials.gov/ was queried on May 18, 2019 for active clinical trials. The following filters were applied: “Phase I”, “Phase II”, “Phase III”, “Recruiting”, “Active but not recruiting”, “Enrolling by invitation”, and “Interventional studies”. Using the phrase, “Pancreatic Cancer,” a total of 481 interventional trials were identified. The date was locked-in, so that any changes in the trials subsequent to that date are not reflected in this study. 41 trials focused on pancreatic neuroendocrine tumors (NETs) and 10 non-therapeutic trials (including imaging and detection) identified through this search were excluded, yielding 430 relevant trials. Note that some studies tested multiple components across trial phases (e.g., phase I/II study). Not all phase I studies are required for registration on https://clinicaltrials.gov/ as they do not all meet the definition of an “applicable clinical trial”, so the list of the earliest trials compiled herein may not be complete [40].

The panoply of returned ‘hits’ was curated and analyzed to best understand and present the state of clinical trials in pancreatic cancer. Data elements were specifically extracted from the https://clinicaltrials.gov/ webpages for each trial, and included: the phase of trial, line of therapy, stage of disease targeted, estimated or targeted accrual size, type of therapeutic intervention, and biologic mechanism of action of the intervention (Supplementary Table 3). Trials with multiple arms testing combinations of novel therapies are separated for some analyses into multiple study interventions. Proposed or accepted biologic mechanisms of action for each intervention were determined from the literature or summarized from classic teachings in cancer biology. In instances where therapies were linked to multiple different biologic mechanisms, the most likely or well characterized mechanism of action was recorded.

Phase III trials represent the most advanced development stage in experimental therapeutics. If positive, phase III results can lead to FDA approvals, alter standard-of-care, and change the treatment paradigm for pancreatic cancer in the near-term. Therefore, these trials were explored more deeply to better understand which advances in this late stage of development were particularly ‘promising’. To better answer this question, we examined interim reports of these trials, as well as associated earlier phase trials of the index intervention. We created an Impact Score (IS) to rank these trials. This strategy enabled a more objective way to estimate the promise and potential impact of the 37 phase II/III and phase III trials. Criteria prioritized interventions which were designed to impact the greatest proportion of pancreatic cancers (i.e., a targeted therapy against a low frequency mutation would receive a lower score), the intended impact (i.e., an intervention designed to improve survival was credited more than one designed to improve pain), and current trial status (an active trial was considered more promising than a negative trial with mature results) (Table 5).

**Table 5:** Phase III trial impact score (IS)

**A. Novelty** (definitions explained below)	
Novel therapeutic mechanism in PDAC^*^	5
Novel drug or indication in PDAC^**^	2
Any PDAC therapy beyond standard of care^***^	1
Established PDAC Intervention^****^	0
**B. Promise**	
Phase III success in other cancer type	+2
Currently under investigation, or has not been tested in other cancers	0
Any phase failure in other cancer type	−2
**C. Allocation** (design used while obtaining available PFS or OS data)	
Randomized	+1
Not applicable	0
Non-randomized	−1
**D. Progression Free Survival** (preceding study or interim results)	
> 6-month improvement	2
1–6-month improvement	1
< 1-month improvement	0
**E. Overall Survival** (preceding study or interim results)	
> 6-month improvement	2
1-6-month improvement	1
< 1-month improvement	0
**F. Expected percentage of pancreatic cancers susceptible^¶^**	
Applicable to > 30% of PDAC tumors	3
Applicable to 5–30% of PDAC tumors	2
Applicable to < 5% of PDAC tumors	1
**G. Desired impact**	
Directly treat PDAC	3
Procedural or sequencing modifications	1
Mitigate symptoms or sequelae	0
**H. Trial Status**	
Active	0
Terminated and published	−5

Emphasis was placed on trials testing novel therapies that anticipate a relatively larger survival benefit. The maximum score a trial may receive is 18. ^a^The intervention under investigation has a mechanism which has never been tested in a PDAC trial beyond the early staged investigations that led to the index trial. That is, the trial is testing a therapy with a novel mechanism in PDAC for the first time. ^b^The compound being tested is novel to PDAC, but there may be other drugs in the same class, or with the same mechanism of action, that have been or are currently under investigation in other PDAC trials. ^c^The studied therapy is not novel to PDAC (due to previous trial failures in studies conducted independently from the index trial), but is not currently FDA approved as a primary treatment option. ^d^The intervention under investigation is already approved and regularly used in PDAC. ^¶^For the purpose of this ranking criteria, and counter to the tenets of precision medicine, the higher the percentage of PDAC cases that are vulnerable, the greater the potential impact.

## SUPPLEMENTARY MATERIALS



## Abbreviations

PDAC: Pancreatic ductal adenocarcinoma
FDA: Food and Drug Administration
FOLFIRINOX: folinic acid, fluorouracil, irinotecan, and oxaliplatin
5-FU: 5-fluorouracil
NCI: National Cancer Institute
NIH: National Institutes of Health
PanCAN: Pancreatic Cancer Action Network
EGFR: epidermal growth factor receptor
PDGFR: platelet-derived growth factor receptor
VEGF: vascular endothelial growth factor
HH: hedgehog
Hsp90: heat shock protein 90
GSK-3: glycogen synthase kinase 3
TGFβ: transforming growth factor beta
PARP: poly-ADP ribose polymerase
FOLFOX: folinic acid, fluorouracil, oxaliplatin
MSI-H: microsatellite instability high
NETs: pancreatic neuroendocrine tumors
