# Supplementary material for: A comprehensive analysis of clinical trials in pancreatic cancer: what is coming down the pike?
Source: Oncotarget. 2020 Sep 22;11(38):3489–501. doi: 10.18632/oncotarget.27727 (PMC7517959; doi:10.18632/oncotarget.27727)
Supplement: Supplementary file 1 [file oncotarget-11-3489-s001.pdf]

A comprehensive analysis of clinical trials in pancreatic cancer: what is coming down the pike?

SUPPLEMENTARY MATERIALS

Supplementary Table 1: Impact score breakdown of each individual phase III trial

Clinical Trials Identifier	Intervention	A	B	C	D	E	F	G	H	TOTAL
NCT03377491	**Tumor Treating Fields** + Gemcitabine + Nab-Paclitaxel (PANOVA-3)	5	+2	−1	1	2	3	3	0	15
NCT03504423	**Devimistat (CPI-613)** + mFFX (AVENGER 500)	5	0	−1	1	2	3	3	0	13
NCT03126435	**EndoTAG-1** + Gemcitabine	2	0	+1	1	1	3	3	0	11
NCT03766295	**Mastinib** + Gemcitabine	2	0	+1	0	1	3	3	0	10
NCT02184195	**Olaparib** (POLO)	5	+2	+1	1	0	2	3	−5	9
NCT02948309	**Mistletoe Extract**	1	0	+1	N/A	1	3	3	0	9
NCT01926197	mFOLFIRINOX +/– **SBRT** in Locally Advanced PDAC	0	+2	−1	N/A	2	3	3	0	9
NCT01077427	**Hyperthermic** Gemcitabine and Cisplatin (HEAT)	1	+2	−1	N/A	1	2	3	0	8
NCT03251365	**Hyperthermic Intra-abdominal Chemotherapy (HIPEC)**	1	+2	0	N/A	N/A	2	3	0	8
NCT03649035	**HybridTherm Probe (HTP)**	1	+2	−1	N/A	0	3	3	0	8
NCT03398291	**Simultaneous Resection** of Pancreatic Cancer and Liver Oligometastasis	1	+2	0	N/A	1	3	1	0	8
NCT03721744	**2nd Line Napabucasin** + Gemcitabine + Nab-Paclitaxel	1	0	−1	0	1	3	3	0	7
NCT02923921	**Pegilodecakin (AM0010)** + FOLFOX	5	0	+1	0	0	3	3	−5	7
NCT03468335	**2nd line Irinotecan Liposomal Injection (Onivyde)**	0	0	−1	1	1	3	3	0	7
NCT01964430	**Adjuvant Gemcitabine + Paclitaxel** vs Gemcitabine (APACT)	0	0	+1	N/A	1	2	3	0	7
NCT02993731	**1st Line Napabucasin** + Gemcitabine + Nab-Paclitaxel	5	0	−1	0	1	3	3	−5	6
NCT03610100	**Acelarin**	5	0	−1	1	N/A	3	3	−5	6
NCT03257033	**Intra-arterial Gemcitabine (RenovoCath)** vs Intra-venous Gemcitabine	0	0	−1	N/A	1	3	3	0	6
NCT02201381	**Metformin + Atorvastatin + Doxycycline + Mebendazole** (METRICS)	1	0	0	N/A	N/A	2	3	0	6
NCT01954992	**Glufosfamide** vs 5-FU	1	−2	+1	N/A	0	3	3	0	6

NCT01013649	Adjuvant Gemcitabine +/– **Erlotinib +/– Radiation + Capecitabine/ Fluorouracil**	0	0	+1	N/A	0	2	3	0	6
NCT02539537	**Neoadjuvant FOLFIRINOX** vs Gemcitabine (NEOPAN)	0	0	−1	1	1	3	1	0	5
NCT02853474	**Early Palliative Care** (metastatic)	0	+2	0	N/A	N/A	3	0	0	5
NCT03472833	**High-dose Vitamin D**	1	0	0	N/A	N/A	3	0	0	4
NCT01827553	**Chemoradiation** vs. Chemotherapy alone in Locally Advanced PDAC (CONKO-7)	0	0	0	N/A	N/A	3	1	0	4
NCT02195232	**Isoquercetin**	1	0	0	N/A	N/A	3	0	0	4
NCT02404363	**Clopidogrel**	2	0	0	N/A	N/A	3	3	−5	3
NCT02919787	**Neoadjuvant** Chemotherapy	0	0	0	N/A	N/A	2	1	0	3
NCT02172976	**Neoadjuvant + Adjuvant FOLFIRINOX** vs Adjuvant Gemcitabine vs (NEPAFOX)	0	0	0	N/A	N/A	2	1	0	3
NCT02506842	**2nd line Gemcitabine + Nab-paclitaxel vs FOLFOX**	0	0	0	N/A	N/A	2	1	0	3
NCT02457156	**Blumgart Anastomosis vs Cattell-Warren Anastomosis**	0	0	0	N/A	N/A	2	1	0	3
NCT02514928	**Resection of Nerve Plexus on Right Half of Celiac and SMA** Associated With Extended Pancreatoduodenectomy	0	0	0	N/A	N/A	2	1	0	3
NCT02871804	**Combined vs Separated Resection of Splenic Vein**	0	0	0	N/A	N/A	2	1	0	3
NCT03269994	**Piperacillin-tazobactam or Cefoxitin** post-surgery	0	0	0	N/A	N/A	2	0	0	2
NCT03434678	**Epidural**	0	0	0	N/A	N/A	2	0	0	2
NCT02340728	**Radiofrequency Ablation** + Self Expandable Metal Stents	1	0	0	N/A	N/A	3	0	−5	0
NCT02349412	**Early Palliative Care**	0	+2	0	N/A	N/A	3	0	−5	0

Supplementary Table 2: Retrospective impact score breakdown of landmark trials for reference

Clinical Trials Identifier	Intervention	A	B	C	D	E	F	G	H	TOTAL
NCT00112658	1st-line **FOLFIRINOX** vs Gemcitabine (mPDAC)	2	+2	+1	1	1	3	3	0	13
NCT00094653	**Ipilimumab +/– gp100 vaccine** (advanced melanoma)	5	0	+1	1	1	3	3	0	14

We assumed that both trials were still active and that the PDAC criteria apply to melanoma.

Supplementary Table 3: Summary of tracked data fields and definitions

Data Element	Definition
**Trial Phase**	FDA-defined stages of clinical testing
Phase I	- Trials that focus on the safety of a drug in a small sample size
Phase II	- Trials that focus on drug efficacy against certain conditions. They are often randomized.
Phase III	- Trials that gather additional information about safety and effectiveness in larger sample sizes. They are randomized.
**Line of Therapy**	Sequence of interventions used to treat disease
First-Line	- Primary, standard treatment for a given disease
Second-Line	- Treatment given after first-line therapy fails
**Stage of Disease**	Extent of cancer
Advanced/Metastatic	- Cancer that has spread beyond the primary organ site
Localized	- Cancer confined to original organ site
Resectable	- Able to safely remove entire tumor by surgery and achieve complete tumor clearance
Borderline	- Not clearly resectable or unresectable (due to partial encasement of local major vasculature). Incomplete tumor clearance, even at a microscopic level, is likely.
Unresectable	- Unable to safely remove tumor by surgery (due to encasement of local major vasculature) and achieve complete tumor clearance. Macroscopic residual disease is likely.
**Type of Intervention**	Action taken to prevent or treat a malady
Novel Drug	- New drug or drug indication that is not normally used in pancreatic cancer patients
Conventional Therapy	- Mainstream treatment that is accepted and used regularly
Gene Therapy	- Introduce specific genetic material to treat disease
Cell Therapy	- Cells are transplanted to induce therapeutic effect
Nutraceutical	- Food or nutritional supplement that may have therapeutic value
Imaging/Detection	- Used to find tumor and to track treatment
Procedure	- Related to surgical resection or other technical manipulation of tumor
Pain/Quality of Life	- Intervention specifically improves patient experience
**Mechanism of Action**	Describes how intervention produces the effect in the body
Immune System	- Stimulates (or suppresses) immune system to better attack the cancer
Cell Signaling	- Interact with specific signaling pathways to interfere with cancer growth and survival
Angiogenesis	- Targets the formation of new blood vessels for nutrients
Metabolism	- Target metabolic pathways of tumor
Hormone Receptors	- Modulate hormone receptors to induce cellular changes
Cell Cycle	- Modulate cell division machinery
DNA Damage Specific	- Specifically interferes with DNA replication or maintenance
Apoptosis Specific	- Induces programmed cell death in cancer cells
Metastasis/Invasion	- Prevent the spread of cancer to other parts of body
Target Symptoms	- Mitigate sequelae associated with disease, but not disease itself
Unknown Mechanism	- Mechanism not yet understood or able to be found in literature
